# Benchmarking emergency department prediction models with machine learning and public electronic health records

**DOI:** 10.1038/s41597-022-01782-9

**Published:** 2022-10-27

**Authors:** Feng Xie, Jun Zhou, Jin Wee Lee, Mingrui Tan, Siqi Li, Logasan S/O Rajnthern, Marcel Lucas Chee, Bibhas Chakraborty, An-Kwok Ian Wong, Alon Dagan, Marcus Eng Hock Ong, Fei Gao, Nan Liu

**Affiliations:** 1 Centre for Quantitative Medicine and Programme in Health Services and Systems Research, Duke-NUS Medical School, Singapore, Singapore; 2 Institute of High Performance Computing, Agency for Science, Technology and Research (A*STAR), Singapore, Singapore; 3 School of Electrical and Electronic Engineering, Nanyang Technological University, Singapore, Singapore; 4 Faculty of Medicine, Nursing and Health Sciences, Monash University, Victoria, Australia; 5 Department of Statistics and Data Science, National University of Singapore, Singapore, Singapore; 6 Department of Biostatistics and Bioinformatics, Duke University, Durham, NC USA; 7 Division of Pulmonary, Allergy, and Critical Care Medicine, Duke University, Durham, NC USA; 8 Department of Emergency Medicine, Beth Israel Deaconess Medical Center, Harvard Medical School, Boston, MA USA; 9 MIT Critical Data, Laboratory for Computational Physiology, Institute for Medical Engineering and Science, Massachusetts Institute of Technology, Cambridge, MA USA; 10 Department of Emergency Medicine, Singapore General Hospital, Singapore, Singapore; 11 SingHealth AI Health Program, Singapore Health Services, Singapore, Singapore; 12 Institute of Data Science, National University of Singapore, Singapore, Singapore

**Keywords:** Health care, Medical research

## Abstract

The demand for emergency department (ED) services is increasing across the globe, particularly during the current COVID-19 pandemic. Clinical triage and risk assessment have become increasingly challenging due to the shortage of medical resources and the strain on hospital infrastructure caused by the pandemic. As a result of the widespread use of electronic health records (EHRs), we now have access to a vast amount of clinical data, which allows us to develop prediction models and decision support systems to address these challenges. To date, there is no widely accepted clinical prediction benchmark related to the ED based on large-scale public EHRs. An open-source benchmark data platform would streamline research workflows by eliminating cumbersome data preprocessing, and facilitate comparisons among different studies and methodologies. Based on the Medical Information Mart for Intensive Care IV Emergency Department (MIMIC-IV-ED) database, we created a benchmark dataset and proposed three clinical prediction benchmarks. This study provides future researchers with insights, suggestions, and protocols for managing data and developing predictive tools for emergency care.

## Introduction

Emergency Departments (ED) experience large volumes of patient flows and growing resource demands, particularly during the current COVID-19 pandemic^1^. This growth has caused ED crowding^2^ and delays in care delivery^3^, resulting in increased morbidity and mortality^4^. Prediction models^5–9^ provide opportunities for identifying high-risk patients and prioritizing limited medical resources. ED prediction models center on risk stratification, which is a complex clinical judgment based on factors such as patient’s likely acute course, availability of medical resources, and local practices^10^.

The widespread use of Electronic Health Records (EHR) has led to the accumulation of large amounts of data, which can be used to develop predictive models to improve emergency care^11–14^. Based on a few large-scale EHR databases, such as Medical Information Mart for Intensive Care III (MIMIC-III)^15^, eICU Collaborative Research Database^16^, and Amsterdam University Medical Centers Database (AmsterdamUMCdb)^17^, several prediction benchmarks have been established^18–20^. These public benchmarks standardized the process of transforming raw EHR data into readily usable data to construct prediction models. They have provided clinicians and methodologists with easily accessible and high-quality medical data, accelerating research and validation efforts^21,22^. These non-proprietary databases and open-source pipelines make it possible to reproduce and improve clinical studies in ways that would otherwise not be possible^18^. While there are some publicly available benchmarks, most pertain to intensive care settings, and there are no widely accepted clinical prediction benchmarks related to the ED. An ED-based public benchmark dataset would lower the entry barrier for new researchers, allowing them to focus on developing novel research ideas.

Machine learning has seen tremendous advances in recent years and has gained increasing popularity in the realm of ED-based prediction models^23–30^. These prediction models involve machine learning, deep learning, interpretable machine learning, and others. However, we have found that researchers often develop an ad-hoc model for one clinical prediction task at a time, using only one dataset^23–28^. There is a lack of comparative studies among different methods and models to predict the same ED outcome, undermining the generalizability of any single model. Generally, existing prediction models were developed on retrospective data without prospective validation in real-world clinical settings. Hence, there remains a need for prospective, comparative studies on accuracy, interpretability, and utility of risk models for ED. Using an extensive public EHR database, we aimed to standardize data preprocessing and establish a comprehensive ED benchmark dataset alongside comparable risk prediction models for three ED-based outcomes. It is expected to facilitate reproducibility and model comparison and accelerate progress toward utilizing machine learning in future ED-based studies.

In this paper, we proposed a public benchmark suite for the ED using a large EHR dataset and introduced three ED-based outcomes: hospitalization, critical outcomes, and 72-hour ED reattendance. We implemented and compared several popular methods for these clinical prediction tasks. We used data from the publicly available MIMIC IV Emergency Department (MIMIC-IV-ED) database^31,32^, which contains over 400,000 ED visit episodes from 2011 to 2019. Our code is open-source (https://github.com/nliulab/mimic4ed-benchmark) so that anyone with access to MIMIC-IV-ED can follow our data processing steps, create benchmarks, and reproduce our experiments. This study provides future researchers with insights, suggestions, and protocols to process the raw data and develop models for emergency care in an efficient and timely manner.

## Methods

This section consists of three parts. First, we describe raw data processing, benchmark data generation, and cohort formation. Second, we introduce baseline models for three prediction tasks. Finally, we elaborate on the experimental setup and model performance evaluation.

### Master data generation

**Fig. 1:**
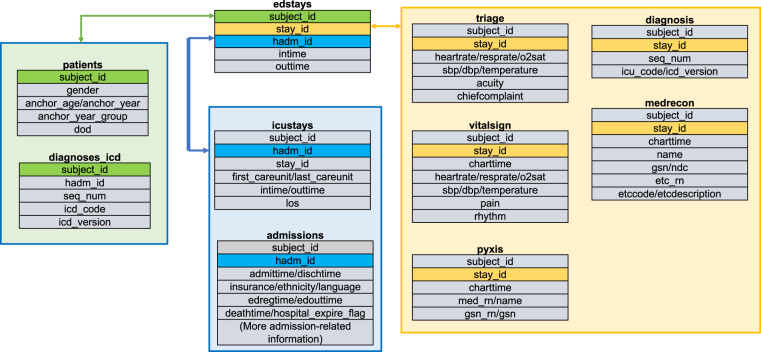
Raw data and the linkage through four unique identifiers (omit *.csv* for table name).

We use standardized terminologies as follows. Patients are referred to by their *subject_id*. Each patient has one or more ED visits, identified by *stay_id* in *edstays.csv*. If there is an inpatient stay following an ED visit, this *stay_id* could be linked with an inpatient admission, identified by *hadm_id* in *edstays.csv*. *subject_id* and *hadm_id* can also be traced back to the MIMIC-IV^31^ database to follow the patient throughout inpatient or ICU stay and patients’ future or past medical utilization, if needed. In the context of our tasks, we used *edstays.csv* as the root table and *stay_id* as the primary identifier. As a general rule, we have one *stay_id* for each prediction in our benchmark tasks. All raw tables were linked through *extract_master_dataset.ipynb*, illustrated in Fig. 1. The linkage was based on the root table, and merged through different identifiers, including *stay_id* (ED), *subject_id*, *hadm_id*, or *stay_id* (ICU). We extracted all high-level information and consolidated them into a master dataset (*master_dataset.csv*).

To construct the master dataset, we reviewed a number of prominent ED studies^5,7,33–35^ to identify relevant variables and outcomes. Moreover, we consulted clinicians and informaticians familiar with the raw data and ED operation to identify and confirm all ED-relevant variables. We excluded variables that were irrelevant, repeated, or largely absent. A list of high-level constructed variables is presented in Supplementary eTable 1, including patient history, variables collected at triage and during ED stay, and primary ED-relevant outcomes. The final master dataset includes 448,972 ED visits by 216,877 unique patients.

### Data processing and benchmark dataset generation

**Fig. 2:**
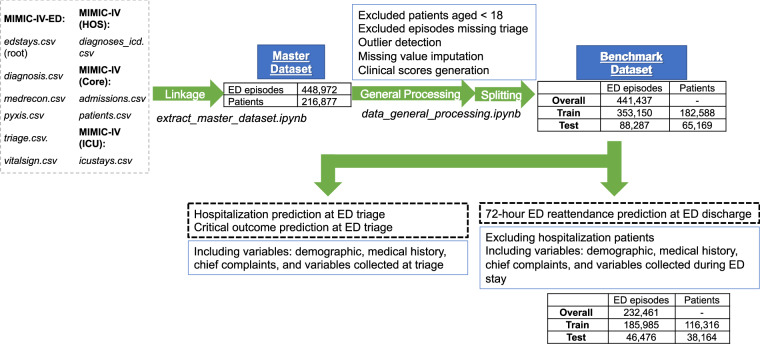
The workflow of data processing from raw data.

The data processing workflow *(data_general_processing.ipynb)*, illustrated in Fig. 2, begins with the master dataset generated previously to derive the benchmark dataset. In the first step, we filtered out all ED visits with patients under 18 years old and those without primary emergency triage class assignments. A total of 441,437 episodes remained after the filtering process.

The raw EHR data cannot be directly used for model building due to missing values, outliers, duplicates, or incorrect records caused by system errors or clerical mistakes. We addressed these issues with several procedures. For vital signs and lab tests, a value would be considered an outlier and marked as missing if it was outside the plausible physiological range as determined by domain knowledge, such as a value below zero or a SpO_2_ level greater than 100%. We followed the outlier detection procedure used in MIMIC-EXTRACT^20^, a well-known data processing pipeline for MIMIC-III. We utilized the thresholds available in the source code repository of Harutyunyan *et al*., where one set of upper and lower thresholds was used for filtering outliers. Any value that falls outside this range was marked as missing. Another set of thresholds was introduced to indicate the physiologically valid range, and any value that falls beyond this range was replaced by its nearest valid value. These thresholds were suggested by clinical experts based on domain knowledge.

For benchmarking purposes, we fixed a test set of 20% (n = 88,287) of ED episodes, covering 65,169 unique patients. Future researchers are encouraged to use the same test set for model comparisons and to interact with the test set as infrequently as possible. The training set consisted of the remaining 80% of ED episodes. The validation set can be derived from the training set if needed. Missing values (including outliers marked as missing and those initially absent) were imputed. In this project, we used the median values from the training set and other options are provided through our code repository. The same values were used for imputation on the test set.

### ICD codes processing

In MMIC-IV, each hospital admission is associated with a group of ICD diagnosis codes (in *diagnoses_icd.csv*), indicating the patients’ comorbidities. We embedded the ICD codes within a time range (e.g., five years) from each ED visit into Charlson Comorbidity Index (CCI)^36^ and Elixhauser Comorbidity Index (ECI)^37^ according to the mapping proposed by Quan H *et al*.^38^. We adopted the codebase from Cates *et al*. and developed the neural network-based embedding with similar network structures to Med2Vec^39^.

### Benchmark tasks

Following are three ED-relevant clinical outcomes. They are all of utmost importance to clinicians and hospitals due to their immense implications on costs, resource prioritization, and patients’ quality of life. Accurate prediction of these outcomes with the aid of big data and artificial intelligence has the potential to transform health services.

- The hospitalization outcome is met with an inpatient care site admission immediately following an ED visit^40–42^. Patients who transitioned to ED observation were not considered hospitalized unless they were eventually admitted to the hospital. As hospital beds are limited, this outcome indicates resource utilization and may facilitate resource allocation efforts. The hospitalization outcome also suggests patient acuity, albeit in a limited way, since hospitalized patients represent a broad spectrum of disease severity.
- The critical outcome^34^ is compositely defined as either inpatient mortality^43^ or transfer to an ICU within 12 hours. This outcome represents the critically ill patients who require ED resources urgently and may suffer from poorer health outcomes if care is delayed. Predicting the critical outcome at ED triage may enable physicians to allocate ED resources efficiently and intervene on high-risk patients promptly.
- The ED reattendance outcome refers to a patient’s return visit to ED within 72 hours after their previous discharge from the ED. It is a widely used indicator of the quality of care and patient safety and is believed to represent patients who may not have been adequately triaged during their first emergency visit^44^.

### Baseline methods

Various triage systems, including clinical judgment, scoring systems, regression, machine learning, and deep learning, were applied to the benchmark dataset and evaluated on each benchmark task, as detailed in Table 1. A five-level triage system, Emergency Severity Index (ESI)^45^, was assigned by a registered nurse based on clinical judgments. Level 1 is the highest priority, and level 5 is the lowest. Several scoring systems were also calculated, including the Modified Early Warning Score (MEWS)^46^, National Early Warning Score (NEWS, versions 1 and 2)^47^, Rapid Emergency Medicine Score (REMS)^48^, and Cardiac Arrest Risk Triage (CART)^49^. It is important to note that there are no neurological features (i.e., Glasgow Coma Scale) in the MIMIC-IV-ED dataset, which may lead to incomplete scores. Three machine learning methods – logistic regression (LR), random forest (RF), and gradient boosting (GB) – were benchmarked as well as deep learning methods multilayer perceptron (MLP)^50^, Med2Vec^39^, and long short-term memory (LSTM)^51–53^. These neural network structures are illustrated in Supplementary eFigure 1. We used the scikit-learn package^54^ with the default parameters for machine learning methods and Keras^55^ for deep learning methods. In addition, the interpretable machine learning method, AutoScore^56–59^, was implemented with its R software package^60^.

**Table 1:** Description of various baseline methods.

	Description	Variables	Hyperparameters	Package used
Traditional machine learning				
Logistic regression (LR)	Use the logistic function to model binary outcomes	Vitals, chief complaints, comorbidities, and age	penalty = ‘l2’, C = 1.0, max_iter = 100	scikit-learn Python package
Random forest (RF)	Build many decision trees in parallel and combine the results through ensemble learning		N_estimators = 100	
Gradient boosting (GB)	Build a number of decision trees in stages and combine the results along the way		Loss = ‘deviance’, learning_rate = 0.1, n_estimators = 100	
Traditional clinical scoring systems				
Emergency Severity Index (ESI)	A subjective five-level triage system assigned by a registered nurse	*triage_acuity*	None	None
Clinical Score: NEWS, NEWS2, MEWS, REMS, CART	Widely used clinical score for risk stratification at ED triage	Vitals, comorbidities, and age	None; No training is needed	None
Interpretable machine learning				
AutoScore	Interpretable machine learning automatic clinical score generator	Vitals, chief complaints, comorbidities, and age	Number of variables, tuned through performance-based parsimony plot	AutoScore R package
Deep learning				
Multilayer perceptron (MLP)	The neural networks of multiple fully connected neurons	Vitals, chief complaints, comorbidities, and age	activation = ‘relu’, learning_rate = 0.001, batch_size = 200, epochs = 20, loss = binary_crossentropy, optimizer = Adam	Keras Python package
Med2Vec	Embedding ICD codes with neural network	Vitals, chief complaints, comorbidities, age and ICD codes in the past 5 years	activation = ‘relu’, learning_rate = 0.001, batch_size = 200, epochs = 100, loss = binary_crossentropy, optimizer = Adam	
LSTM	A special type of RNN which is capable of learning long-term dependencies	Basic static variables, and temporal variables of vital signs collected in the ED	activation = ‘relu’, learning_rate = 0.001, batch_size = 200, epochs = 20, loss = binary_crossentropy, optimizer = Adam	

CART: Cardiac Arrest Risk Triage.

LSTM: Long short-term memory.

MEWS: Modified Early Warning Score.

NEWS: National Early Warning Score.

NEWS: National Early Warning Score, Version 2.

REMS: Rapid Emergency Medicine Score.

RNN: Recurrent neural network.

### Experiments, settings, and evaluation

We conducted all experiments on a server equipped with an Intel Xeon W-2275 processor, 128GB of memory, and an Nvidia RTX 3090 GPU, and the running time at model training was recorded. Deep learning models were trained using the Adam optimizer and binary cross-entropy loss. The AutoScore method optimized the number of variables through a parsimony plot. As the implementation was only for demonstration purposes, Module 5 of the clinical fine-tuning process in AutoScore was not implemented. We conducted the receiver operating characteristic (ROC) and precision-recall curve (PRC) analysis to evaluate the performance of all prediction models. The area under the ROC curve (AUROC) and the area under the PRC (AUPRC) values were reported as an overall measurement of predictive performance. Model performance was reported on the test set, and 100 bootstrapped samples were applied to calculate 95% confidence intervals (CI). Furthermore, we computed the sensitivity and specificity measures under the optimal cutoffs, defined as the points nearest to the upper-left corner of the ROC curves.

## Results

### Baseline characteristics of the benchmark dataset

We compiled a master dataset comprising 448,972 ED visits of 216,877 unique patients. After excluding incomplete or pediatric visits, a total of 441,437 adult ED visits were finally included in the benchmark dataset. They were randomly split into 80% (353,150) training data and 20% (88,287) test data. Table 2 and Supplementary eTable 2 summarize the baseline characteristics of the entire cohort, stratified by outcomes. The average age of the patients was 52.8 years old, and 54.3% (n = 239,794) of them were females. Compared with other patients, those with critical outcomes displayed higher body temperature and heart rate, and were prescribed a greater amount of medication. Additionally, they were more likely to have fluid and electrolyte disorders, coagulopathy, cancer, cardiac arrhythmias, valvular disease, and pulmonary circulation disorders.

**Table 2:** Basic characteristics of the benchmark dataset. Continuous variables are presented as *mean (SD)*; binary or categorical variables are presented as *count (%);* more variables are described in Supplementary eTable 2.

	Overall	Outcomes			
		Hospitalization outcome		Critical outcomes	72-hour ED reattendance
		Discharge	Hospitalized		
# Emergency visits	441,437	232,461	208,976	26,174	15,299
*Demographic*					
Age	52.80 (20.60)	46.29 (19.36)	60.03 (19.50)	65.43 (17.85)	50.40 (18.70)
Gender					
Female	239794 (54.3%)	133874 (57.6%)	105920 (50.7%)	12168 (46.5%)	7068 (46.2%)
Male	201643 (45.7%)	98587 (42.4%)	103056 (49.3%)	14006 (53.5%)	8231 (53.8%)
Emergency Severity Index					
Level 1	25363 (5.7%)	5349 (2.3%)	20014 (9.6%)	8888 (34.0%)	462 (3.0%)
Level 2	147178 (33.3%)	45445 (19.5%)	101733 (48.7%)	14099 (53.9%)	3838 (25.1%)
Level 3	237565 (53.8%)	151843 (65.3%)	85722 (41.0%)	3176 (12.1%)	9849 (64.4%)
Level 4	30160 (6.8%)	28704 (12.3%)	1456 (0.7%)	11 (0.0%)	1091 (7.1%)
Level 5	1171 (0.3%)	1120 (0.5%)	51 (0.0%)	0 (0.0%)	59 (0.4%)
*Chief complaints*					
Chest pain	30756 (7.0%)	13790 (5.9%)	16966 (8.1%)	1107 (4.2%)	907 (5.9%)
Abdominal pain	50868 (11.5%)	25801 (11.1%)	25067 (12.0%)	1711 (6.5%)	1961 (12.8%)
Headache	16601 (3.8%)	11967 (5.1%)	4634 (2.2%)	620 (2.4%)	627 (4.1%)
Shortness of breath	1285 (0.3%)	402 (0.2%)	883 (0.4%)	213 (0.8%)	24 (0.2%)
Back pain	17625 (4.0%)	12369 (5.3%)	5256 (2.5%)	282 (1.1%)	621 (4.1%)
Cough	9269 (2.1%)	5293 (2.3%)	3976 (1.9%)	411 (1.6%)	244 (1.6%)
Nausea/vomiting	10666 (2.4%)	5606 (2.4%)	5060 (2.4%)	466 (1.8%)	401 (2.6%)
Fever/chills	15267 (3.5%)	4651 (2.0%)	10616 (5.1%)	1427 (5.5%)	398 (2.6%)
Syncope	8198 (1.9%)	4409 (1.9%)	3789 (1.8%)	359 (1.4%)	167 (1.1%)
Dizziness	10928 (2.5%)	6337 (2.7%)	4591 (2.2%)	365 (1.4%)	287 (1.9%)
*Information collected at triage*					
Temperature (Celsius)	36.71 (0.54)	36.68 (0.49)	36.75 (0.59)	36.75 (0.66)	36.69 (0.51)
Mean arterial pressure (mmHg)	96.59 (14.86)	97.55 (13.84)	95.51 (15.86)	92.08 (17.86)	97.91 (14.77)
Heart rate (bpm)	85.05 (17.46)	83.90 (16.32)	86.32 (18.56)	90.74 (20.93)	87.07 (16.94)
Respiratory rate (bpm)	17.57 (2.49)	17.30 (2.11)	17.87 (2.83)	18.91 (4.32)	17.42 (2.16)
Oxygen saturation (%)	98.40 (2.42)	98.80 (2.00)	97.95 (2.75)	97.30 (3.70)	98.39 (2.51)
Systolic blood pressure (mmHg)	134.84 (22.14)	135.14 (20.67)	134.51 (23.67)	129.17 (26.21)	135.09 (21.79)
Diastolic blood pressure (mmHg)	77.46 (14.71)	78.76 (13.76)	76.01 (15.57)	73.53 (16.46)	79.33 (14.62)
Pain scale	4.15 (3.60)	4.67 (3.58)	3.58 (3.54)	3.08 (3.02)	4.74 (3.78)

The outcome statistics for the benchmark data are presented in Table 3, demonstrating a balanced stratification of the training and test data. In the overall cohort, 208,976 (47.34%) episodes require hospitalization, 26,174 (5.93%) episodes have critical outcomes, and 15,299 (3.47%) result in 72-hour ED reattendance.

**Table 3:** Outcome statistics of prediction tasks. The number of ED visits and their proportions in training and test data are shown for each outcome subgroup.

	Outcome				
	Hospitalization	ICU transfer in 12 hours	Inpatient mortality	Critical outcome	ED reattendance in 72 hours
Training data	167165 (47.34%)	19791 (5.60%)	3295 (0.93%)	21048 (5.96%)	12365 (3.50%)
Test data	41811 (47.36%)	4816 (5.45%)	796 (0.90%)	5126 (5.80%)	2934 (3.32%)
Total	208976 (47.34%)	24607 (5.57%)	4091 (0.93%)	26174 (5.93%)	15299 (3.47%)

### Variable importance and ranking

Following a descending order of variable importance obtained from RF, the top 10 variables selected for each predictive task are presented in Table 4. Vital signs show significant predictive value in all three tasks. Age is also among the top predictive variables for all tasks, underscoring the impact of aging on emergency care utilization. While the triage level (i.e., ESI) is highly related to the hospitalization and critical outcome, it is not relevant to 72-hour ED reattendance. Conversely, despite its lower importance for hospitalization and critical outcomes, ED length of stay becomes the top variable for 72-hour ED reattendance prediction. The previous health utilization variable seems to be a less important feature for ED-based tasks.

**Table 4:** Top 10 variables from each benchmark task based on random forest variable importance.

Hospitalization		Critical outcomes		72-hour ED reattendance	
Variable	Importance	Variable	Importance	Variable	Importance
Age (years)	0.1266	Age (years)	0.09980	Age (years)	0.0840
ESI at triage	0.1118	Systolic BP at triage (mmHg)	0.09978	ED length of stays (hours)	0.0837
Systolic BP at triage (mmHg)	0.0872	Heart rate at triage (bpm)	0.0932	Systolic BP at ED (mmHg)	0.0789
Heart rate at triage (bpm)	0.0853	ESI at triage	0.0921	Diastolic BP at ED (mmHg)	0.0767
Diastolic BP at triage (mmHg)	0.0828	Diastolic BP at triage (mmHg)	0.0838	Heart rate at ED (bpm)	0.0762
Temperature at triage (Celsius)	0.0784	Temperature at triage (Celsius)	0.0766	Temperature at ED (Celsius)	0.0669
Pain scale at triage	0.0469	Respiratory rate at triage (bpm)	0.0567	Counts of medication reconciliation	0.0518
Oxygen saturation at triage (%)	0.0425	Oxygen saturation at triage (%)	0.0505	Pain scale at triage	0.0439
Respiratory rate at triage (bpm)	0.0402	Pain scale at triage	0.0398	Counts of medication reconciliation	0.0393
Hospitalizations in the past year	0.0276	ED visits in the past year	0.0187	Oxygen saturation at ED (%)	0.0387

BP: Blood pressure.

ED: Emergency department.

ESI: Emergency Severity Index.

### Benchmark task evaluation

**Fig. 3:**
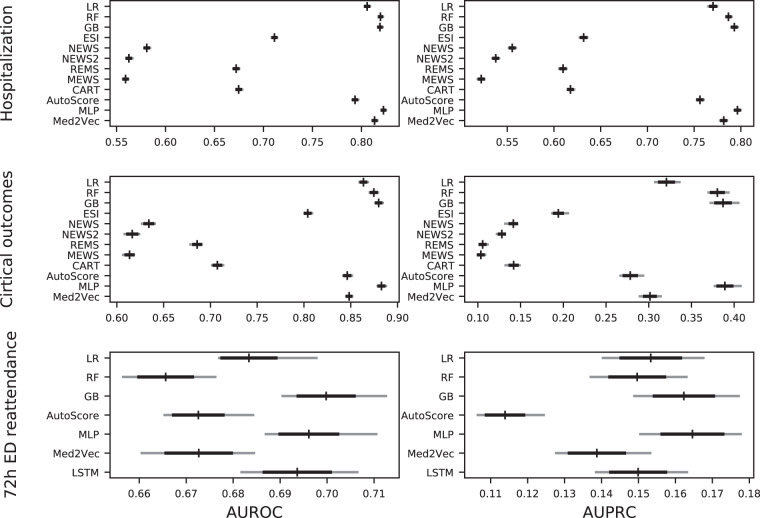
Bar plots comparing the performance of various prediction models based on three different outcomes. AUROC: The area under the receiver operating characteristic curve. AUPRC: The area under the precision-recall curve. CART: Cardiac Arrest Risk Triage. ESI: Emergency Severity Index. GB: Gradient boosting. LSTM: Long short-term memory. LR: Logistic regression. MEWS: Modified Early Warning Score. MLP: Multilayer perceptron. NEWS: National Early Warning Score. NEWS2: National Early Warning Score, Version 2. REMS: Rapid Emergency Medicine Score. RF: Random forest.

Machine learning exhibited a higher degree of discrimination in predicting all three outcomes. Gradient boosting achieved an AUC of 0.880 (95% CI: 0.876–0.884) for the critical outcome and an AUC of 0.819 (95% CI: 0.817–0.822) for the hospitalization outcome. However, the corresponding performance for 72-hour ED reattendance was considerably lower. Compared with gradient boosting, deep learning could not achieve even higher performance. While traditional scoring systems did not show good discriminatory performance, interpretable machine learning-based AutoScore achieved an AUC of 0.846 (95% CI: 0.842–0.851) for critical outcomes with seven variables, and 0.793 (95% CI: 0.791–0.797) for hospitalization outcomes with 10 variables. Tables 5–7 and Supplementary eTable 3 present the performance of of a variety of machine learning and scoring systems on different prediction tasks assessed by various metrics on the test set. Moreover, they are also plotted in Fig. 3.

**Table 5:** Comparison of the performance of different models for hospitalization prediction at triage.

Model	AUROC (95% CI)	AUPRC (95% CI)	Threshold	Sensitivity (95% CI)	Specificity (95% CI)	Runtime*	Number of variables
LR	0.806 (0.803–0.809)	0.770 (0.765–0.775)	0.446	0.747 (0.722–0.749)	0.721 (0.719–0.745)	3.715	64
RF	0.819 (0.819–0.822)	0.787 (0.785–0.790)	0.490	0.754 (0.742–0.767)	0.734 (0.724–0.747)	58	64
GB	0.819 (0.817–0.822)	0.793 (0.790–0.797)	0.474	0.754 (0.736–0.759)	0.729 (0.727–0.752)	60	64
ESI	0.711 (0.709–0.714)	0.632 (0.628–0.636)	2	0.582 (0.578–0.586)	0.784 (0.781–0.787)	N/A^a^	1
NEWS	0.581 (0.579–0.584)	0.555 (0.552–0.559)	1	0.565 (0.561–0.570)	0.540 (0.537–0.544)	N/A	6
NEWS2	0.563 (0.560–0.566)	0.538 (0.534–0.541)	1	0.519 (0.514–0.522)	0.563 (0.559–0.567)	N/A	6
REMS	0.672 (0.669–0.675)	0.610 (0.605–0.613)	3	0.714 (0.709–0.716)	0.564 (0.559–0.568)	N/A	6
MEWS	0.559 (0.557–0.562)	0.522 (0.518–0.526)	2	0.300 (0.296–0.302)	0.810 (0.808–0.813)	N/A	6
CART	0.675 (0.673–0.678)	0.618 (0.615–0.622)	4	0.702 (0.698–0.706)	0.586 (0.582–0.592)	N/A	4
AutoScore	0.793 (0.791–0.797)	0.756 (0.753–0.760)	45	0.722 (0.717–0.749)	0.721 (0.698–0.725)	N/A	10
MLP	0.822 (0.821–0.825)	0.796 (0.793–0.800)	0.457	0.757 (0.745–0.767)	0.734 (0.724–0.746)	171	64
Med2Vec	0.813 (0.812–0.816)	0.782 (0.778–0.785)	0.431	0.744 (0.738–0.748)	0.731 (0.728–0.739)	1044	64 + 7930^#^

AUROC: The area under the receiver operating characteristic.

AUPRC: The area under the precision-recall curve.

CART: Cardiac Arrest Risk Triage.

CI: Confidence interval.

ESI: Emergency Severity Index.

GB: Gradient boosting.

LSTM: Long short-term memory.

LR: Logistic regression.

MEWS: Modified Early Warning Score.

MLP: Multilayer perceptron.

NEWS: National Early Warning Score.

NEWS2: National Early Warning Score, Version 2.

REMS: Rapid Emergency Medicine Score.

RF: Random forest.

*The unit of the running time in seconds.

^a^Runtime calculation is not applicable for clinical scores (including AutoScore), as their development usually involves some manual processes.

^#^The dataset contains 7930 distinct ICD codes.

**Table 6:** Comparison of the performance of different models for critical outcomes prediction at triage.

Model	AUROC (95% CI)	AUPRC (95% CI)	Threshold	Sensitivity (95% CI)	Specificity (95% CI)	Runtime	Number of variables
LR	0.864 (0.859–0.868)	0.321 (0.308–0.336)	0.064	0.783 (0.773–0.809)	0.785 (0.756–0.793)	4	64
RF	0.875 (0.870–0.879)	0.380 (0.370–0.393)	0.078	0.803 (0.794–0.810)	0.792 (0.791–0.795)	51	64
GB	0.880 (0.876–0.884)	0.387 (0.373–0.405)	0.064	0.809 (0.790–0.821)	0.790 (0.783–0.810)	58	64
ESI	0.804 (0.801–0.809)	0.194 (0.187–0.205)	2	0.870 (0.863–0.875)	0.640 (0.637–0.643)	N/A^a^	1
NEWS	0.634 (0.627–0.640)	0.141 (0.132–0.144)	2	0.464 (0.453–0.472)	0.795 (0.793–0.798)	N/A	6
NEWS2	0.616 (0.608–0.623)	0.128 (0.122–0.131)	2	0.410 (0.399–0.586)	0.823 (0.531–0.824)	N/A	6
REMS	0.686 (0.679–0.691)	0.105 (0.102–0.111)	5	0.681 (0.668–0.687)	0.616 (0.613–0.619)	N/A	6
MEWS	0.613 (0.606–0.618)	0.103 (0.100–0.108)	2	0.430 (0.417–0.439)	0.770 (0.768–0.772)	N/A	6
CART	0.707 (0.701–0.713)	0.141 (0.132–0.148)	6	0.590 (0.578–0.598)	0.731 (0.728–0.733)	N/A	4
AutoScore	0.846 (0.842–0.851)	0.278 (0.267–0.293)	66	0.804 (0.784–0.810)	0.728 (0.726–0.747)	N/A	7
MLP	0.883 (0.879–0.888)	0.389 (0.377–0.407)	0.046	0.813 (0.805–0.829)	0.787 (0.772–0.794)	171	64
Med2Vec	0.848 (0.845–0.851)	0.301 (0.290–0.314)	0.004	0.783 (0.768–0.798)	0.767 (0.756–0.788)	1052	64 + 7930^#^

^a^Runtime calculation is not applicable for clinical scores (including AutoScore), as their development usually involves some manual processes.

^#^The dataset contains 7930 distinct ICD codes.

**Table 7:** Comparison of the performance of different models for 72-hour ED reattendance prediction at ED disposition.

Model	AUROC (95% CI)	AUPRC (95% CI)	Threshold	Sensitivity (95% CI)	Specificity (95% CI)	Runtime	Number of variables
LR	0.683 (0.677–0.698)	0.153 (0.140–0.168)	0.041	0.627 (0.604–0.652)	0.636 (0.630–0.653)	2	67
RF	0.666 (0.657–0.676)	0.150 (0.137–0.163)	0.060	0.540 (0.531–0.605)	0.706 (0.620–0.708)	29	67
GB	0.700 (0.691–0.713)	0.162 (0.149–0.177)	0.038	0.639 (0.607–0.672)	0.642 (0.617–0.679)	30	67
AutoScore	0.673 (0.665–0.684)	0.114 (0.107–0.124)	27	0.621 (0.596–0.637)	0.628 (0.622–0.665)	N/A^a^	12
MLP	0.696 (0.687–0.710)	0.165 (0.151–0.178)	0.027	0.644 (0.628–0.667)	0.641 (0.631–0.648)	91	67
Med2Vec	0.673 (0.661–0.684)	0.139 (0.128–0.153)	0.002	0.574 (0.562–0.621)	0.682 (0.635–0.691)	538	67 + 7930^#^
LSTM	0.694 (0.682–0.706)	0.150 (0.139–0.163)	0.034	0.630 (0.606–0.663)	0.650 (0.623–0.686)	11423	67^

^a^Runtime calculation is not applicable for clinical scores (including AutoScore), as their development usually involves some manual processes.

^Include 7 temporal variables.

^#^The dataset contains 7930 distinct ICD codes.

## Discussion

This paper proposes standardized data benchmarks for future researchers who are interested in analyzing large-scale ED-based clinical data. Our study provides a pipeline to process raw data from the newly published MIMIC-IV-ED database and generates a benchmark dataset, the first of its kind in the ED context. The benchmark dataset contains approximately half a million ED visits, and is highly accessible by researchers who plan to replicate our experiments or further build upon our work. Additionally, we demonstrated several clinical prediction models (e.g., machine learning and clinical scoring systems) on routinely available information using this benchmark dataset for three ED-relevant outcomes: hospitalization, critical outcome, and ED reattendance. Our benchmark dataset also supports linkage to the main MIMIC-IV database, allowing researchers to analyze a patient’s clinical course from the time of ED presentation through the hospital stay.

Our study showed that machine learning models demonstrated higher predictive accuracy, consistent with the previous studies^9,19,61^. Complex deep learning^62^ models such as Med2Vec and LSTM did not perform better than simpler models. These results suggest that overly complex models do not necessarily improve performance with relatively low-dimensional ED data. Furthermore, predictions made by black-box machine learning have critical limitations in clinical practice^63,64^, particularly for decision-making in emergency care. Although machine learning models outperform in terms of predictive accuracy, the lack of explainability makes it challenging for frontline physicians to understand how and why the model reaches a particular conclusion. In contrast, scoring systems combine just a few variables using simple arithmetic and have a more explicit clinical representation^56^. This transparency allows doctors to understand and trust model outputs more easily and contributes to the validity and acceptance of clinical scores in real-world settings^65,66^. In our experiments, predefined scoring systems were unable to achieve satisfactory accuracy. However, AutoScore-based data-driven scoring systems complemented them with much higher accuracy while maintaining the advantages of the point-based scores^7^.

The primary goals of ED prediction models are to identify high-risk patients accurately and to allocate limited resources efficiently. While physicians can generally determine the severity of a patient’s acute condition, their decisions necessarily contain subjective influences that depend on the healthcare context and practitioner’s knowledge. Objective predictive systems can outperform expert intuition^40^ in making multi-criteria decisions by taking away interpersonal variation between healthcare practitioners^41^. This could be a potentially valuable tool for emergency physicians who have to constantly multitask^67^, especially in the complex ED environment where decisions must be made based on heuristics and dynamic changes^68^. This study explores data-driven methods to provide an objective assessment for three ED-relevant risk triaging tasks based on large-scale public EHRs. Several previous studies^34,69,70^ have also demonstrated that objective electronic predictive triage systems provide more accurate differentiation for patients with regards to clinical outcomes compared with traditional subjective clinical assessment. In addition, the openly accessible nature of the models makes them suitable for reproducibility and improvement. The scientific research community can make full use of the benchmark data and the prediction benchmark in future research.

Three ED-based clinical outcomes were explored in this study with clinical significance. Accurate prediction of those three outcomes could help optimize ED resources with timely care delivery and mitigate ED delayed care problems. Our hospitalization prediction model can give an idea of the likelihood of hospitalization at the time of triage to the patients and staff, even before a physician is assigned to examine the patient^40,41^. Identifying patients who might end up with critical illness or death could potentially differentiate high-risk patients from more-stable patients and efficiently allocate finite ED resources^5–7^. Predicting ED reattendance could also allow providers to reconsider patient’s discharge plans and provide optimal care for those who had been prematurely discharge^71^. In addition, these three outcomes are interrelated yet represent distinct groups of predictors. Prediction models of hospitalization and critical outcomes share a similar set of predictors, whereas ED reattendances depend on various other variables. Although understanding personal risk or prognosis has great value, it is more important to realize the full potential of these prediction models in improving emergency care in clinical practice. In the future, focus should be shifted to filling the implementation gap by considering the model’s actionability and real-world utility^72^.

From a data science perspective, this study contributes to the scientific community by standardizing research workflows and reducing barriers of entry^18^ for both clinicians and data scientists engaged in ED research. In the future, researchers may use this data pipeline to process raw MIMIC-IV-ED data. They may also develop new models and evaluate them against our ED-based benchmark tasks and prediction models. Additionally, our pipeline does not focus exclusively on ED data; we also provide linkages to the MIMIC-IV main database^73–76^ with all ICU and inpatient episodes. Data scientists interested in extracting ED data as additional variables and linking them to the other settings of the MIMIC-IV database can exploit our framework to streamline their research without consulting different ED physicians. With the help of this first large-scale public ED benchmark dataset and data processing pipeline, researchers can conduct high-quality ED research without needing a high level of technical proficiency.

This study has several limitations. First, although the study is based on an extensive database, it is still a single-center study. The performance of different methods used in this study may differ in other healthcare settings. Nevertheless, the proposed clinical prediction pipeline could still be used as a reference for future big data research in ED. Furthermore, examining whether models trained on the benchmark data generalize to other clinical datasets would be interesting. Second, the benchmark dataset established in this study is based on EHR data extracted from the hospital’s patient portal with routinely collected variables, where certain potential risk factors, such as socioeconomic status, critical first look, and neurological features, were not recorded. For example, some health utilization data such as intubation and resuscitation have been proven to be predictive of overall mortality and should have been included in our model. Furthermore, neurological features like the Glasgow Coma Scale (GCS)^77^ score, were not available in the MIMIC-IV-ED database. These features are considered significant predictors in the ED setting and could have greatly increased the performance of our models. In addition, the dataset lacks sufficient information to detect out-of-hospital deaths, which may introduce bias into our predictions. Lastly, simple median imputation was employed to handle missing vital signs in the raw data, potentially obscuring data structures that could have been captured by more sophisticated methods. Future researchers utilizing our data pipeline should attempt to apply more advanced techniques for dealing with missing values. Despite these limitations, the data processing pipeline can be leveraged widely when new researchers wish to conduct ED research using the MIMIC-IV-ED database.

## Supplementary information

Supplementary Materials

## Data Availability

The data that support the findings of this study are available from the MIMIC-IV database^31^: https://physionet.org/content/mimiciv/1.0/ and MIMIC-IV-ED database^32^: https://physionet.org/content/mimic-iv-ed/1.0/.

## Code availability

The code used to analyze the data in the current study is available at: https://github.com/nliulab/mimic4ed-benchmark.
