# Supplementary material for: Benchmarking emergency department prediction models with machine learning and public electronic health records
Source: Sci Data. 2022 Oct 27;9:658. doi: 10.1038/s41597-022-01782-9 (PMC9610299; doi:10.1038/s41597-022-01782-9)
Supplement: Supplementary file 1 — Supplementary Materials [file 41597_2022_1782_MOESM1_ESM.pdf]

Supplementary Materials

eTable 1. List of high-level constructed variables in the master dataset and their origins and categories.

eTable 2. Characteristics of the benchmark dataset with included variables (continued with Table 2 in the main text).

eTable 3. Comparison of performance of different models applied to critical outcomes at ED disposition.

eFigure 1. Neural network structure of MLP, LSTM and Med2Vec.

eTable 1. List of high-level constructed variables in the master dataset and their origins and categories.

Category	Sub-category	Source table (omit *.csv* below)	Variable description	Variable name in the master dataset
Patient history	Past ED visits	*edstays*	ED visits in the past month, ED visits in the past three months, ED visits in the past year	*n_ed_30d, n_ed_90d, n_ed_365d*
	Past hospitalizations	*admissions*	Hospitalizations in the past month, Hospitalizations in the past three months, Hospitalizations in the past year	*n_hosp_30d, n_hosp_90d, n_ hosp_365d*
	Past ICU admissions	*icustays*	ICU admissions in the past month, ICU admissions in the past three months, ICU admissions in the past year	*n_icu_30d, n_icu_90d, n_icu_365d*
	Comorbidities	*diagnoses_icd, d_icd_diagnoses*	Charlson Comorbidity Index (CCI, 17 variables), Elixhauser Comorbidity Index (ECI, 30 variables)	*cci_** (* represents 17 variables), *eci_** (* represents 30 variables)
Information at triage	Demographics	*patients*	Age, Gender	*age, gender*
	Triage-vital signs	*triage*	Emergency Severity Index (ESI) Vital signs collected at triage: Temperature (Celsius), Heart rate (bpm), Oxygen saturation (%), Systolic blood pressure (mmHg), Diastolic blood pressure (mmHg), Pain scale	*triage_acuity triage_temperature, triage_heartrate, triage_o2sat, triage_sbp, triage_dbp, triage_pain*
	Triage-chief complaint	*triage*	Top 10 chief complaints identified in the ED	*chiefcom_** (* represents ten different chief complaints)
Information before ED discharge	ED vital signs	*vitalsigns*	Vital signs collected during ED stay (last measurement): Temperature (Celsius), Heart rate (bpm), Oxygen saturation (%), Systolic blood pressure (mmHg), Diastolic blood pressure (mmHg)	*ed_temperature, ed_heartrate, ed_o2sat, ed_sbp, ed_dbp*
	ED administrative	*edstays*	ED length of stay (hours)	*ed_los*
	Medication reconciliation	*medrecon*	Counts of medication reconciliation	*n_medrecon*
	Medication prescription	*pyxis*	Counts of medication prescription in current ED stay	*n_med*
Outcomes	Hospitalization	*edstays:hadm_id*	Whether the patient is admitted to inpatient stay following the current ED visit	*outcome_ hospitalization*
	Inpatient mortality	*patients:dod admissions:dischtime*	Whether the patient dies in the hospital before discharge	*outcome_inhospital _mortality*
	ICU transfer from ED	*icustays:intime, edstays:outtime*	Whether the patient is transferred to ICU from ED within 12 hours	*outcome_icu_transfe r_12h*
	ED reattendance	*edstays*	Whether the patient revisits ED after the discharge from the index ED visit within three days (72 hours or 3 days)	*outcome_ed_revisit_ 3d*
	Critical outcomes	*master_dataset: outcome_icu_transfer_12h , outcome_inhospital _mortality*	Whether the patient fulfills either inpatient mortality or ICU transfer within 12 hours	*outcome_critical*

* denotes the task-specific wildcard string

eTable 2. Characteristics of the benchmark dataset with included variables (continued with Table 2 in the main text). Continuous variables are presented as *mean (SD)*; binary or categorical variables are presented as *count (%)*.

	**Overall**	**Outcomes**			
		**Hospitalization outcome**		**Critical outcomes**	**72-hour ED reattendance**
		**Discharge**	**Hospitalized**		
# Emergency visits	441,437	232,461	208,976	26,174	15,299
Comorbidities (Charlson Comorbidity Index)					
Myocardial infarction	24773 (5.6%)	6487 (2.8%)	18286 (8.8%)	2807 (10.7%)	1080 (7.1%)
Congestive heart failure	40784 (9.2%)	10253 (4.4%)	30531 (14.6%)	5191 (19.8%)	1285 (8.4%)
Peripheral vascular disease	21985 (5.0%)	5706 (2.5%)	16279 (7.8%)	2614 (10.0%)	658 (4.3%)
Stroke	21104 (4.8%)	6431 (2.8%)	14673 (7.0%)	2392 (9.1%)	745 (4.9%)
Dementia	7387 (1.7%)	2039 (0.9%)	5348 (2.6%)	889 (3.4%)	252 (1.6%)
Chronic pulmonary disease	62610 (14.2%)	23142 (10.0%)	39468 (18.9%)	5361 (20.5%)	3115 (20.4%)
Rheumatoid disease	9115 (2.1%)	3013 (1.3%)	6102 (2.9%)	774 (3.0%)	273 (1.8%)
Peptic ulcer disease	8315 (1.9%)	2306 (1.0%)	6009 (2.9%)	902 (3.4%)	318 (2.1%)
Liver disease					
None	402913 (91.3%)	220993 (95.0%)	181920 (87.1%)	22516 (86.0%)	12695 (83.0%)
Mild liver disease	29645 (6.7%)	9489 (4.1 %)	20156 (9.6 %)	2586 (9.9 %)	2153 (14.1%)
Moderate/severe liver disease	8879 (2.0%)	1979 (0.9 %)	6900 (3.3 %)	1072 (4.1 %)	451 (2.9%)
Diabetes					
None	355132 (80.5%)	204810 (88.2%)	150322 (72.0%)	18038 (68.9%)	11591 (75.8%)
Diabetes without chronic complications	58375 (13.2%)	19874 (8.5%)	38501 (18.4%)	5231 (20.0%)	2649 (17.3%)
Diabetes with complications	27930 (6.3%)	7777 (3.3%)	20153 (9.6%)	2905 (11.1%)	1059 (6.9%)
Hemiplegia	5085 (1.2%)	1573 (0.7%)	3512 (1.7%)	659 (2.5%)	177 (1.2%)
Moderate to severe chronic kidney disease	42952 (9.7%)	11060 (4.8%)	31892 (15.3%)	4736 (18.1%)	1263 (8.3%)
Cancer					
None	401805 (91.0%)	222186 (95.6%)	179619 (85.9%)	21580 (82.4%)	14195 (92.8%)
Local tumor, leukemia, and lymphoma	28631 (6.5%)	7746 (3.3%)	20885 (10.0%)	3122 (11.9%)	842 (5.5%)
Metastatic solid tumor	11001 (2.5%)	2529 (1.1%)	8472 (4.1%)	1472 (5.6%)	262 (1.7%)
AIDS	4079 (0.9%)	1578 (0.7%)	2501 (1.2%)	258 (1.0%)	426 (2.8%)
Elixhauser Comorbidity Index					
Cardiac arrhythmias	61501 (13.9%)	18815 (8.1%)	42686 (20.4%)	6601 (25.2%)	2746 (17.9%)
Valvular disease	22464 (5.1%)	6210 (2.7%)	16254 (7.8%)	2654 (10.1%)	702 (4.6%)
Pulmonary circulation disorders	20357 (4.6%)	5607 (2.4%)	14750 (7.1%)	2564 (9.8%)	739 (4.8%)
Hypertension, uncomplicated	44612 (10.1%)	11542 (5.0%)	33070 (15.8%)	5023 (19.2%)	1344 (8.8%)
Hypertension, complicated	107846 (24.4%)	39697 (17.1%)	68149 (32.6%)	8282 (31.6%)	5214 (34.1%)
Other neurological disorders	33515 (7.6%)	11194 (4.8%)	22321 (10.7%)	3251 (12.4%)	2292 (15.0%)
Hypothyroidism	29407 (6.7%)	9900 (4.3%)	19507 (9.3%)	2645 (10.1%)	963 (6.3%)
Lymphoma	4832 (1.1%)	1253 (0.5%)	3579 (1.7%)	469 (1.8%)	112 (0.7%)

Coagulopathy	31206 (7.1%)	8389 (3.6%)	22817 (10.9%)	3776 (14.4%)	1597 (10.4%)
Obesity	39138 (8.9%)	14919 (6.4%)	24219 (11.6%)	2886 (11.0%)	1525 (10.0%)
Weight loss	23216 (5.3%)	6448 (2.8%)	16768 (8.0%)	2609 (10.0%)	1216 (7.9%)
Fluid and electrolyte disorders	82782 (18.8%)	25384 (10.9%)	57398 (27.5%)	8381 (32.0%)	4199 (27.4%)
Blood loss anemia	6044 (1.4%)	1699 (0.7%)	4345 (2.1%)	699 (2.7%)	258 (1.7%)
Deficiency anemia	26437 (6.0%)	8626 (3.7%)	17811 (8.5%)	2402 (9.2%)	1384 (9.0%)
Alcohol abuse	34542 (7.8%)	12501 (5.4%)	22041 (10.5%)	2207 (8.4%)	3731 (24.4%)
Drug abuse	29648 (6.7%)	11538 (5.0%)	18110 (8.7%)	1480 (5.7%)	3036 (19.8%)
Psychoses	12536 (2.8%)	4766 (2.1%)	7770 (3.7%)	603 (2.3%)	1185 (7.7%)
Depression	72698 (16.5%)	27630 (11.9%)	45068 (21.6%)	4725 (18.1%)	4192 (27.4%)
Information collected during ED stay					
Temperature (Celsius)	36.76 (0.37)	36.72 (0.32)	36.80 (0.42)	36.85 (0.61)	36.73 (0.37)
Heart rate (bpm)	78.14 (14.38)	76.25 (12.84)	80.24 (15.65)	87.49 (20.14)	79.97 (13.85)
Respiratory rate (bpm)	17.25 (2.47)	16.92 (1.87)	17.60 (2.96)	19.29 (4.55)	17.03 (1.87)
Oxygen saturation (%)	98.19 (2.94)	98.55 (2.83)	97.79 (3.01)	97.58 (3.83)	98.19 (2.90)
Systolic blood pressure (mmHg)	127.39 (19.50)	127.62 (18.56)	127.13 (20.49)	122.37 (22.23)	128.72 (19.50)
Diastolic blood pressure (mmHg)	73.56 (13.56)	75.49 (12.68)	71.42 (14.17)	67.95 (15.14)	75.97 (13.47)
Counts of medication prescription in the ED	2.91 (3.30)	1.79 (2.24)	4.15 (3.81)	5.33 (4.30)	2.70 (3.21)
Counts of medication reconciliation	6.11 (6.77)	4.44 (5.88)	7.96 (7.20)	7.80 (7.53)	5.17 (6.59)
ED length of stays (h)	4.78 (7.47)	0.30 (0.40)	9.75 (8.41)	5.62 (5.18)	4.20 (7.83)

eTable 3. Comparison of performance of different models applied to critical outcomes at ED disposition.

**Critical Outcomes prediction at ED disposition**							
Model	AUROC (95% CI)	AUPRC (95% CI)	Threshold	Sensitivity (95% CI)	Specificity (95% CI)	Runtime*	Number of variables
LR	0.859 (0.855-0.865)	0.362 (0.347-0.374)	0.058	0.775 (0.762-0.791)	0.783 (0.767-0.793)	3	67
RF	0.932 (0.930-0.935)	0.565 (0.554-0.583)	0.1	0.856 (0.861-0.880)	0.850 (0.836-0.852)	47	67
GB	0.934 (0.932-0.936)	0.557 (0.543-0.571)	0.077	0.857 (0.847-0.861)	0.850 (0.846-0.857)	68	67
MLP	0.937 (0.935-0.939)	0.559 (0.544-0.572)	0.069	0.88 (0.865-0.884)	0.837 (0.837-0.848)	168	67
LSTM	0.945 (0.943-0.947)	0.597 (0.583-0.608)	0.070	0.868 (0.867-0.888)	0.866 (0.85-0.868)	21781	200
ESI	0.804 (0.799-0.808)	0.194 (0.187-0.201)	2	0.870 (0.861-0.875)	0.640 (0.637-0.643)	N/A[a]	1
NEWS	0.634 (0.627-0.640)	0.141 (0.132-0.147)	2	0.464 (0.452-0.476)	0.795 (0.793-0.798)	N/A	6
NEWS2	0.616 (0.610-0.623)	0.128 (0.121-0.135)	2	0.410 (0.399-0.592)	0.823 (0.532-0.825)	N/A	6
REMS	0.686 (0.682-0.691)	0.105 (0.101-0.11)	5	0.681 (0.671-0.689)	0.616 (0.613-0.618)	N/A	6
MEWS	0.613 (0.608-0.619)	0.103 (0.099-0.109)	2	0.430 (0.419-0.438)	0.770 (0.768-0.772)	N/A	6
CART	0.707 (0.701-0.713)	0.141 (0.135-0.15)	6	0.590 (0.576-0.598)	0.731 (0.728-0.733)	N/A	4

CART: Cardiac Arrest Risk Triage
ESI: Emergency Severity Index
GB: Gradient boosting
LR: Logistic regression
LSTM: Long short-term memory
MEWS: Modified Early Warning Score
MLP: Multilayer perceptron
NEWS: National Early Warning Score
REMS: Rapid Emergency Medicine Score
RF: Random forest
* The unit of the running time in seconds.
[a] Runtime calculation is not applicable for clinical scores (including AutoScore), as their development usually involves many manual processes.

eFigure 1. Neural network structure of MLP, LSTM and Med2Vec.

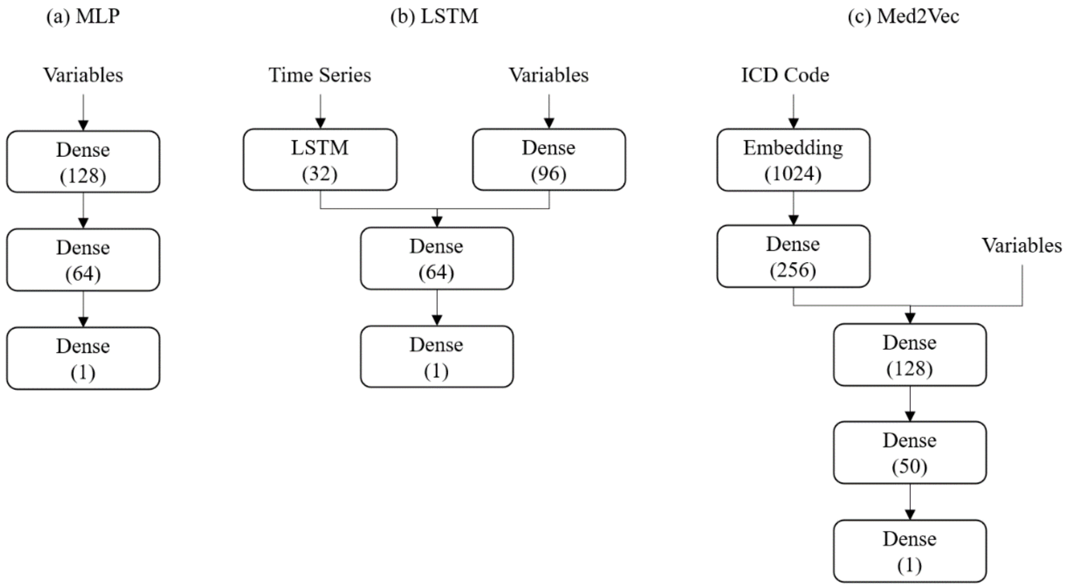

LSTM: Long short-term memory
MLP: Multilayer perceptron
